# A third HSAN5 mutation disrupts the nerve growth factor furin cleavage site

**DOI:** 10.1177/1744806918809223

**Published:** 2018-10-08

**Authors:** Samiha S Shaikh, Michael S Nahorski, C Geoffrey Woods

**Affiliations:** 1 Cambridge Institute for Medical Research, Addenbrooke's Biomedical Research Centre, Cambridge, UK; 2 Department of Clinical Genetics, Addenbrooke's Hospital, Cambridge, UK

**Keywords:** Nerve growth factor, mutation, furin cleavage, HSAN5

## Abstract

Bi-allelic dysfunctional mutations in nerve growth factor (NGF) cause the rare human phenotype hereditary sensory and autonomic neuropathy type 5 (HSAN5). We describe a novel NGF mutation in an individual with typical HSAN5 findings. The mutation c.361C>T, p.R121W is at the last residue of the furin cleavage motif Arg-Ser-Lys-Arg in proNGF. We show that the p.R121W mutation completely abolishes the formation of mature NGF-β. Surprisingly, mutant p.R121W cells produced very little proNGF. Instead, the two progressive cleavage products of proNGF were produced, proA-NGF and proB-NGF, with proB-NGF being the predominant NGF-derived peptide and the only peptide secreted by mutant p.R121W cells. We found that the ability of the p.R121W mutation to cause tropomyosin receptor kinase A autophosphorylation and mitogen-activated protein kinase phosphorylation was significantly reduced compared to controls (p < 0.05 and p < 0.01). By studying the PC12 cell line morphology and neurite length over a week, we found the p.R121W mutation had residual, but much reduced, neurotrophic activity when compared to wild-type NGF. Finally, we assessed whether the p.R121W mutation affected apoptosis and found a reduced protective effect compared to wild-type NGF. Our results suggest that the p.R121W NGF mutation causes HSAN5 through negating the ability of furin to cleave proNGF to produce NGF-β.

## Introduction

**Figure 1.**
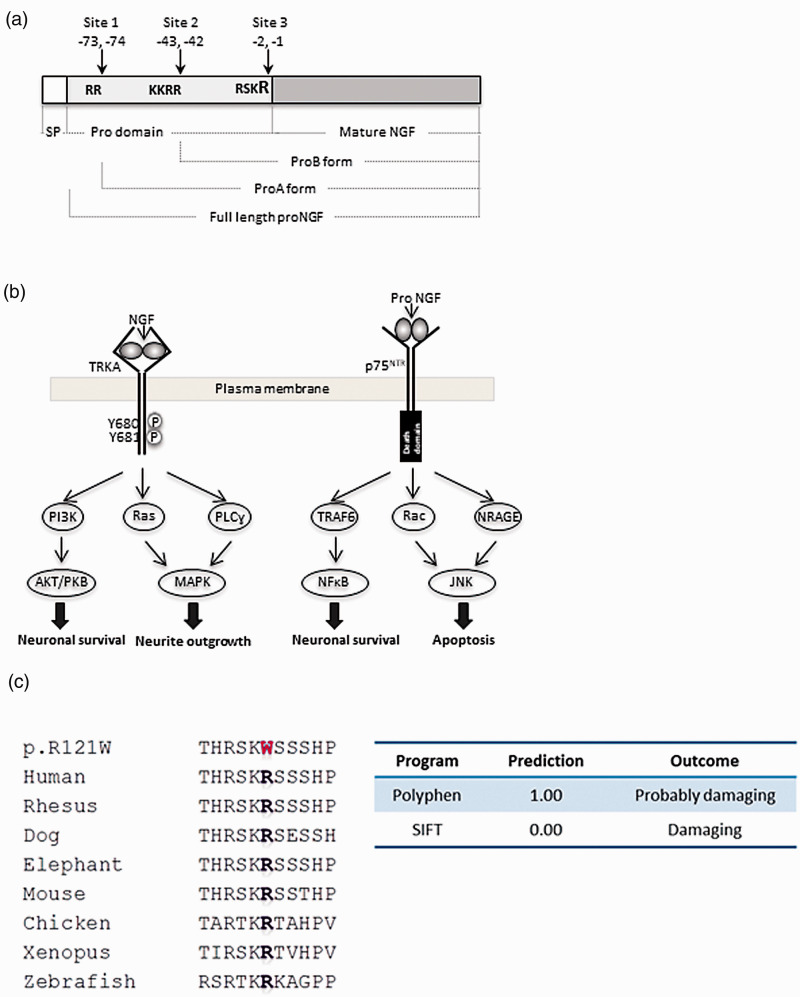
Structure and signalling pathway of NGF and pathogenicity of a novel NGF mutation. (a) NGF is synthesised as pre-proNGF which contains a signal peptide sequence, a prodomain and the mature NGF sequence. After synthesis, the signal peptide is removed to generate full-length proNGF. The prodomain has three cleavage signals. Cleavage at sites 1 and 2 produces the proA and proB forms of proNGF, respectively. Cleavage at site 3 produces mature NGF. The mutation studied in this article, p.R121W, occurs in the last residue of the cleavage motif at site 3. (b) NGF binding to TRKA results in TRKA dimerisation and autophosphorylation. TRKA subsequently phosphorylates Y496 and Y791. Phosphorylation of Y496 leads to activation of the Ras/ERK pathway and the AKT pathway resulting in neuronal differentiation and survival, respectively. Phosphorylation of Y791 results in PLCγ activation and neuronal differentiation. The recruitment of the adaptor protein FRS2 to Y496 results in the formation of signalling endosomes and long-term signalling. NGF-TRKA signalling is augmented by interaction with p75NTR. Signalling of proNGF through binding to p75NTR can cause apoptosis through the recruitment of different members of the Rho GTPase family and subsequent activation of the JNK pathway. JNK activation leads to cell death by activation of the transcription factor c-jun and also activation of caspase 3/9. ProNGF binding to p75NTR can also induce neuronal survival through recruitment of TRAF6 to p75NTR and consequently activation of NFκB. (c) The missense p.R121W mutation occurred in an evolutionary conserved amino acid. The candidate mutation is shown in red. Both Polyphen and sorting intolerant from tolerant predicted the mutation to be pathogenic with highest scores for a probably damaging mutation.

Nerve growth factor (NGF) was the first member of the neurotrophin family to be identified and has since been discovered to have multiple significant roles in pain.^1–3^ The human *NGF* gene is located on chromosome 1.p13.2 and consists of three exons, of which only the third exon is translated to produce the large 35 kDa precursor pre-proNGF. Pre-proNGF has an N-terminal signal peptide that is followed by the prodomain and the mature domain (Figure 1(a)). Cleavage of the signal peptide occurs in the endoplasmic reticulum to yield proNGF, which spontaneously forms non-covalently linked homodimers. The prodomain is cleaved predominantly by furin at the Arg-Ser-Lys-Arg (RSKR) motif located at positions -1 and -2 with respect to the mature NGF sequence to generate mature NGF-β peptide.^4,5^ The prodomain also contains two other cleavage sites RR (-73 and -74) and KKRR (-43 and -44), and cleavage at these sites can generate the processing intermediates proA and proB, respectively (Figure 1(a)). ProA has a size of 26 kDa, and proB has a size of 21 kDa.^6,7^

The major NGF receptor is tropomyosin receptor kinase A (TRKA) encoded by the gene neurotrophic tyrosine kinase receptor type 1 (*NTRK1*). Ligand binding with TRKA induces dimerisation and autophosphorylation and subsequent activation of downstream signalling summarised in Figure 1(b) to promote neuronal survival and differentiation. NGF can also bind to the pan neurotrophic receptor (p75NTR) to augment signalling through TRKA. It has been demonstrated that proNGF interacts with high affinity to p75NTR and to sortilin. The resulting signalling complex recruits adaptor proteins such as NRAGE and Rac to activate the JNK signalling cascades that primarily drive apoptosis.^8–12^ ProNGF binding to p75NTR can also promote the activation of NFκB and thereby induce neuronal survival.^13,14^

Mutations in *NGF* cause the rare autosomal recessive disorder hereditary sensory and autonomic neuropathy type 5 (HSAN5, online mendelian inheritance in man #608654). HSAN5 is characterised by the selective loss of unmyelinated C fibres and myelinated Aδ fibres,^15–17^ lack of pain perception and recurrent injuries as well as a susceptibility towards *Staphylococcus aureus* infections.^18^ To date, two mutations in *NGF* have been identified: p.R221W and p.V232fs.^15,19^ Assessment of these two *NGF* mutations showed that the original mutation, p.R221W, resulted in reduced processing of NGF, whilst the second mutation, p.V232fs, abolished processing and altered neurotrophic activity.^19,20^

We identified a third *NGF* mutation in a patient with typical HSAN5, c.361C>T, corresponding to the change p.R121W. The mutation occurred in the last residue of the motif RSKR recognised by the proprotein convertase furin as a cleavage site (see Figure 1). Functional work was performed to assess the pathogenicity of this mutation and to gain deeper insight into the role of proNGF and NGF in neuronal survival.

## Materials and methods

### Clinical genetics, molecular genetics, sequencing and segregation studies

A young male adult was ascertained with a history of congenital painlessness, lack of sweating, reduced learning abilities compared to other members of his family and the development of multiple Charcot’s joints. The patient had mildly delayed milestones, but his presenting features were repeated minor injuries and unsteadiness by two years of age. Thereafter, it was noted that he seemed to ignore painful injuries. By five years, he was considered to have mild developmental delay, and over the next five years it became apparent that he had static mild cognitive delay. It became clear to the parents that he was painless, rather than having a high pain threshold by seven years. He needed assistance in a normal school but was physically able, healthy and had no autistic features.^21^ He was not noted to sweat after exercise or in hot weather nor to complain of extremes of environmental temperature. He had one episode of osteomyelitis as a teenager. From his teenage years on, his major problem has been the development of Charcot’s joints in his hips, knees and ankles, which caused painless but progressive orthopaedic deformities. His phenotype was compared to other individuals seen by us with HSAN4 and HSAN5, and we considered his phenotype more similar to HSAN4 due to bi-allelic TRKA mutations.^22^

Initially, the genes *NTRK1* and *SCN9A* were sequenced, and when no clear pathogenic mutation was found, exome sequencing was performed.^23^ The exome was analysed as previously described. The *NGF* mutation was assessed by Sanger sequencing of the proband and their parents. Research Ethics was obtained for this study from the Cambridge East UK Medical Research Ethics Committee.

### Cloning or expression constructs

Full-length *NGF* cDNA (NM_002506), including a 5′ *HindIII* and a 3′ *BamHI* restriction sites, was ordered from GeneArt (ThermoFisher Scientific). A C-terminal mCherry-tagged *NGF* was obtained by the introduction of this insert into the pmCherry-N1 vector using a double restriction enzyme approach as described previously.^22^ The point mutation was introduced using the QuickChange II site-directed mutagenesis kit (Agilent Technologies) following the manufacturer’s protocol.

### Cell culture and conditions

PC12 cells were grown in flasks coated with collagen I (Gibco) at 300 µg/ml and were cultured in Roswell Park Memorial Institute (RPMI) 1640-Glutamax (Gibco) supplemented with 10% horse serum (HS), 5% foetal bovine serum (FBS), 100 µg/ml penicillin and 100 µg/ml streptomycin.

For most assays unless otherwise mentioned, PC12 cells were plated at a concentration of 7.6 × 10^5^ cells/ml in collagen-coated 12-well plates. For caspase assays, PC12 cells were seeded on collagen-coated 96-well plates at a concentration of 1.5 × 10^5^ cells/ml. For localisation studies and neurite outgrowth studies, PC12 cells were plated at 7.6 × 10^5^ cells/ml and 2.5 × 10^4^ cells/ml, respectively, on entactin-collagen IV-laminin coated coverslips or plates (5 µg/cm^2^, Millipore).

Cells were transiently transfected with Lipofectamine 2000 (ThermoFisher Scientific) using a transfection ration of 4:1.

### Localisation

To look at the localisation of NGF in undifferentiated cells, 48-h post-transfection, PC12 cells were fixed in 4% paraformaldehyde for 10 min. To look at NGF localisation in differentiated cells, 24 h post-transfection cells were treated with 100 ng/ml mouse NGF (Peprotech) for five days and then fixed. Cells were stained with an mCherry antibody (ThermoFisher Scientific 16D7, 1:1000), and Alexa-546 conjugated secondary antibodies were purchased from Invitrogen (1:1000).

All images were acquired with an LSM710 laser-scanning Meta confocal microscope (Carl Zeiss) using a ×63 oil-immersion objective.

### NGF secretion studies

To determine whether the mutant NGF protein affected secretion, NGF in the media and in the lysate of transfected cells was determined using the human β-NGF DuoSet enzyme-linked immunosorbent assay (ELISA) kit (R&D systems) according to the manufacturer’s protocol.

Forty-eight hours post-transfection, media were removed and cells were washed in Dulbecco’s modified Eagle’s medium (DMEM) supplemented with 0.25% FBS. Cells were then incubated in 550 µl DMEM supplemented with 0.25% FBS for 4 h at 37°C. Media were then collected and spun for 5 min at 13,000 r/min to sediment cell debris. After the removal of media, cells were washed in phosphate-buffered saline, and lysate was harvested in radioimmunoprecipitation assay buffer (RIPA) buffer (Tris pH 7.4, NaCl 150 mM, EDTA, 0.5 mM, 1% Triton), containing protease inhibitors (Roche Applied Sciences). Lysates were cleared by centrifugation at 13,000 r/min at 4°C for 25 min and levels of total cellular protein were tested using the DC protein assay kit (BioRad). Protein estimation was performed prior to NGF ELISA. Moreover, 15 µg lysate and 200 µl conditioned media were used with the ELISA kit in duplicate. Absorbance signals were read on a microplate reader (PerkinElmer) set at 450 nm.

### NGF processing

The different forms of NGF in media and in cell lysate were determined by western blotting. Both media and lysate samples were prepared as described above, but 500 µl of media was concentrated in Amicon Ultra-0.5 centrifugal filter units (Ultracel-30 membrane, Millipore) by centrifugation at 13,000 r/min for 25 min. The reverse spin was performed immediately at 2000 r/min for 2 min.

Lysate and concentrated media were run on 10% Tris-HCl gels (Novex) and transferred to polyvinylidene fluoride membrane (Millipore). Blots were then probed with a monoclonal mCherry antibody (ThermoFisher Scientific 16D7, 1:1000), and β-actin (Abcam ab8226, 1:1000) was used as the loading control. Secondary antibodies were purchased from Dako, and signal was detected using the chemiluminescent horseradish peroxidase substrate (Millipore).

### TRKA autophosphorylation assay

The ability of NGF to induce TRKA autophosphorylation was determined. Conditioned media of tagged wild-type NGF, p.R121W and mCherry were prepared by changing media 24 h post-transfection to 0.5% DMEM and incubating for 24 h. Media were spun to sediment cell debris before use. Although PC12 cells contain endogenous TrkA, the cells were transfected with wild-type NTRK1-green fluorescent protein (GFP), as we wanted to see the interaction of human NGF with human TRKA, and the ELISA kit is specific for human TRKA. Twenty-four hours post-transfection with wild-type TRKA, cells were stimulated for 15 min at 37°C with the pre-prepared conditioned media. Lysate was prepared as described above with one alteration: RIPA buffer was supplemented with a 1:100 dilution of phosphatase inhibitor cocktails 2 and 3 (Sigma).

The autophosphorylation levels and total TRKA levels were determined using the PathScan® Phospho-TRKA Sandwich ELISA 674/675 Kit (CST) and PathScan® Total TRKA Sandwich ELISA kit (CST), following manufacturers’ protocol. The relative levels of phosphorylated TRKA were calculated by dividing phosphorylated values by total TRKA values.

### Mitogen-activated protein kinase assay

Phosphorylated mitogen-activated protein kinase (MAPK) was detected using the PathScan® Phospho-p44/42 MAPK (Thr202/Tyr204) Sandwich ELISA (CST). Briefly, conditioned media were prepared (as described in the above section), and untransfected cells were stimulated with the conditioned media for 15 min at 37°C. Lysate was harvested in RIPA buffer with phosphatase inhibitors, and 15 µg of lysate was loaded in wells and the assay was run following the manufacturer’s protocol.

### Neurotrophic activity

PC12 cells were transfected with the constructs as described above. Forty-eight hours post-transfection, media were removed and changed to RPMI supplemented with 2% HS and 1% FBS. The following day (day 3), cells were imaged from 15 random fields of view, and media were changed again. Another 48 h later (day 5), cells were imaged from 15 random fields of view, and media were changed again. Another 48 h later (day 7), cells were imaged from 15 random fields of view.

Cells were imaged with an EVOS cell microscope (ThermoFisher Scientific) using the ×10 objective. Cells were then analysed using the ImageJ software. The percentage of neurite-bearing cells and total neurite length per cell was quantified. A neurite was identified as being more than twice the cell body length.^24^ For the calculation of average neurite length, all extensions were measured and divided by the total number of cells counted.

### Caspase assay

To look at the effect of NGF on caspase activation and apoptosis, 48 h post-transfection, media were removed and changed to 3% RPMI. After culture for five days, caspase assays were performed using the ApoTox-Glo Triplex assay using the manufacturer’s protocol. The ratio of live cells to dead cells is independent of cell number and therefore caspase values were normalised to this ratio, according to the manufacturer’s recommendation.

### Statistical analysis

The data show the mean ± standard error of the mean. Statistical significance was calculated using a one-way analysis of variance (ANOVA), followed by Tukey’s post hoc test. Significance was set at *p < 0.05, **p < 0.01 or ***p < 0.001.

## Results

### Clinical genetics, molecular genetics, sequencing and segregation studies

The index case phenotype was typical for individuals compared to literature cases and those previously seen by us with HSAN4 and HSAN5.^19,22,25^ Therefore, we had expected either *NTRK1* or *NGF* mutations to be present. Exome analysis revealed a homozygous *NGF* mutation c.361C>T, p.R121W. This was confirmed by Sanger sequencing of the proband and shown to be heterozygous in their clinically unaffected parents. The mutation has not been previously reported, and due to its location within the canonical furin RSKR cleavage site in proNGF, this made it a candidate mutation for further functional assessment.

### Putative mutation in NGF

The homozygous p.R121W was identified in a patient with HSAN5 (numbering based on RefSeq ID NP_002497). There are no variations at this position recorded in the 1000 Genomes Server, dbSNP (http://www.ncbi.nlm.nih.gov/SNP/), the Exome Variant Server (http://evs.gs.washington.edu/EVS/) or the ExAC database (http://exac.broadinstitute.org). The mutation occurred in the last residue of the furin motif, which is an evolutionarily conserved residue and was predicted to be pathogenic by both Polyphen and sorting intolerant from tolerant (SIFT) softwares (Figure 1(c)). A number of different studies were undertaken to determine the pathogenic mechanism behind the mutation.

### Localisation of NGF

**Figure 2.**
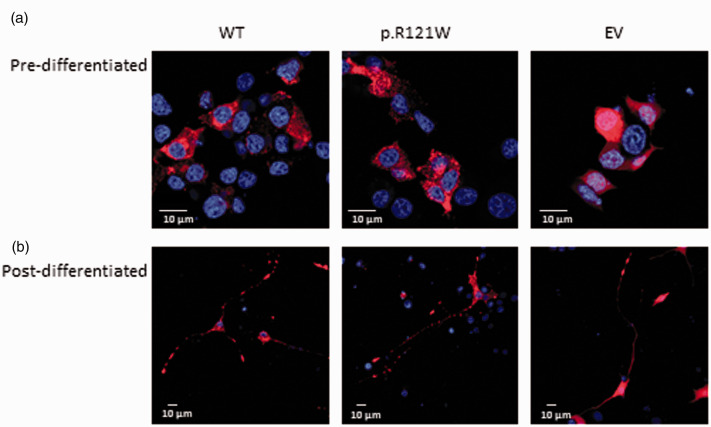
Localisation of wild-type and mutant NGF. (a) Transfected cells were stained against mCherry (red). Wild-type NGF in undifferentiated cells had a vesicular pattern of expression and was localised in the cytoplasm. p.R121W had a similar localisation pattern. Empty vector transfected cells had a uniform expression throughout the cytoplasm. Representative images are shown from three independent repeats. (b) Wild-type NGF in differentiated PC12 cells had a vesicular expression pattern and in addition to being localised in the cytoplasm, it was also present along the axons and neurite tips. A similar expression pattern was seen in p.R121W transfected cells. Empty vector transfected cells had a uniform expression throughout the cell. Representative images are shown from three independent repeats. WT: wild-type; EV: empty vector.

Initially, the localisation of the mutant NGF protein was determined to see whether trafficking was altered. Wild-type NGF was primarily localised in the cytoplasm in a punctate expression pattern (Figure 2). Mutant NGF also had a similar vesicular expression pattern in the cytoplasm, and no obvious aberration in localisation could be seen (Figure 2(a)).

In neurons, NGF is transported along axons in either an anterograde or retrograde manner.^26^ To see if the mutant NGF could be trafficked along axons, transfected PC12 cells were treated with mouse NGF over a period of five days to induce differentiation of naïve cells. Wild-type NGF was located in vesicles in the cytoplasm and along the neurites, and expression was highest at neurite tips (Figure 2(b)). No obvious changes in expression could be seen in p.R121W-expressing cells.

### Secretion of p.R121W NGF

As the localisation of NGF was unaffected, the ability of NGF to be secreted was investigated. Cells were transfected with tagged wild-type and mutant NGF constructs. Media and lysate were collected, and an NGF ELISA was performed on the samples.

**Figure 3.**
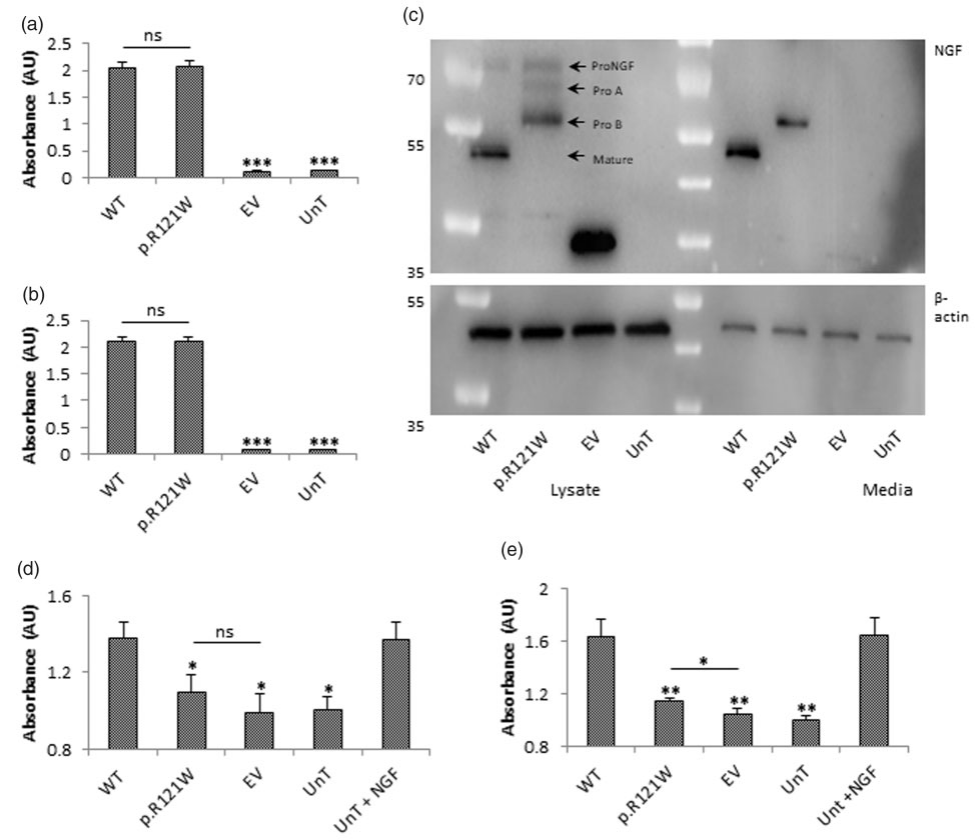
NGF processing and downstream signalling. (a) PC12 cells were transfected and the levels of NGF in the lysate and conditioned media were determined. No statistical difference was found between the NGF levels in lysate of cells transfected with wild-type and mutant NGF. (b) NGF levels in conditioned media produced by cells transfected with wild-type and mutant NGF. No statistical difference was found between the NGF levels in media of cells transfected with wild-type and mutant NGF. Statistical differences between wild-type NGF-treated and the other treatment groups are indicated as ***p<0.001 (one-way ANOVA, followed by Tukey’s post hoc test). (c) To determine the processing events that occur in the wild-type and mutant NGF proteins, the constructs were expressed in PC12 cells. Wild-type NGF was mainly present as mature NGF in lysate and was the only form being secreted. p.R121W was present as the proA and proB form in the lysate and only the proB form as present in the media. No mature NGF was produced. Representative image of n = 3 is shown. (d) PC12 cells transfected with wild-type TRKA were treated with conditioned media for 15 min or with 100 ng/ml NGF as the positive control, and TRKA autophosphorylation levels were determined. The *x-*axis represents wild-type TRKA transfected cells treated with conditioned media from WT NGF, p.R121W and empty vector transfected cells or untransfected cells (UnT) or with exogenous NGF (UnT+NGF). Wild-type NGF conditioned media induced TRKA autophosphorylation to a similar level as the positive control. Treatment of cells with mutant NGF or empty vector conditioned media did not cause any significant induction of TRKA autophosphorylation. (e) Untransfected PC12 cells were treated with conditioned media for 15 min or with 100 ng/ml NGF as the positive control and MAPK phosphorylation levels were determined. The *x-*axis represents untransfected cells treated with conditioned media from WT NGF, p.R121W and empty vector transfected cells or untransfected cells (UnT) or with exogenous NGF (UnT+NGF). Treatment of cells with wild-type conditioned media stimulated MAPK phosphorylation to a similar level as the positive control. Treatment of cells with p.R121W conditioned media resulted in a slight induction of MAPK phosphorylation compared to empty vector-treated cells, but this level was significantly lower than the wild-type NGF-treated response. Statistical differences between cells treated with wild-type NGF conditioned media and other conditioned media treatment groups are indicated as *p<0.05, **p<0.01 (one-way ANOVA, followed by Tukey’s post hoc test). Error bars represent standard error of the mean, n = 3. WT: wild-type; EV: empty vector; UnT: untransfected.

NGF was readily detectable in the lysate of tagged wild-type NGF-transfected cells (Figure 3(a)). The level of NGF in mutant-transfected cells was not significantly different to wild-type levels. The level of NGF in the media of both wild-type and p.R121W NGF-transfected cells was easily detectible and was similar to each other (Figure 3(b)). Thus, it appeared that the mutation did not affect the secretion of NGF. NGF could not be detected in lysate or media of cells transfected with empty vector or in untransfected cells, and in both cases, NGF levels were significantly lower than wild-type NGF-transfected cells (p < 0.001).

### Cleavage of p.R121W NGF

As secretion was not affected, the processing events were investigated. Cell lysate and concentrated media of tagged wild-type and mutant NGF-transfected cells were collected and subject to Western blotting which was probed with an mCherry (tagged-NGF) antibody. Lysate of tagged wild-type NGF-transfected cells showed that there was a faint band at 70 kDa and a main band at about 50 kDa, corresponding to proNGF and mature NGF, respectively. Only mature NGF was found in the media.

Lysate of p.R121W-transfected cells produced no mature NGF. However, three bands were present which corresponded to full-length proNGF and the proA and proB forms of proNGF (Figure 3(c)). Out of all these forms, only the proB form was secreted and present in the media. These results indicate that the mutation prevented full cleavage of proNGF, thereby abolishing the production of mature NGF with only the proB form being secreted.

### Downstream signalling of ProB form

As the proB form could still be secreted, the effects of proNGF on the activation of TRKA and downstream signalling were investigated. One of the first signalling events to follow NGF binding to TRKA is its autophosphorylation, and this was investigated. Human TRKA expressing PC12 cells were treated with conditioned media from tagged wild-type NGF, p.R121W NGF and empty vector-transfected cells. Cells were also treated with media from untransfected cells as well as with 100 ng/ml NGF. Tagged wild-type NGF-treated cells had a relative TRKA autophosphorylation of 1.4 AU, as did the positive control NGF-treated cells (Figure 3(d)). In p.R121W-treated cells, TRKA autophosphorylation was significantly reduced to 1.0 AU (p < 0.05), which was not significantly different from the empty vector-transfected cells and untransfected cells.

Next, the effect of the proB form, generated by the p.R121W mutation, on the downstream MAPK signalling pathway was determined. Induction of MAPK phosphorylation was similar in wild-type-tagged NGF-stimulated cells and untransfected cells stimulated with exogenous NGF (Figure 3(e)). p.R121W resulted in statistically significant stimulation of MAPK phosphorylation compared to empty vector-treated cells (p < 0.05), but this was significantly reduced compared to the response of tagged wild-type NGF-treated cells (p < 0.01) indicating that the proB form resulted in only a small induction of MAPK kinase activation, and significantly less than achieved by mature NGF.

### Neurotrophic activity of proB form

**Figure 4.**
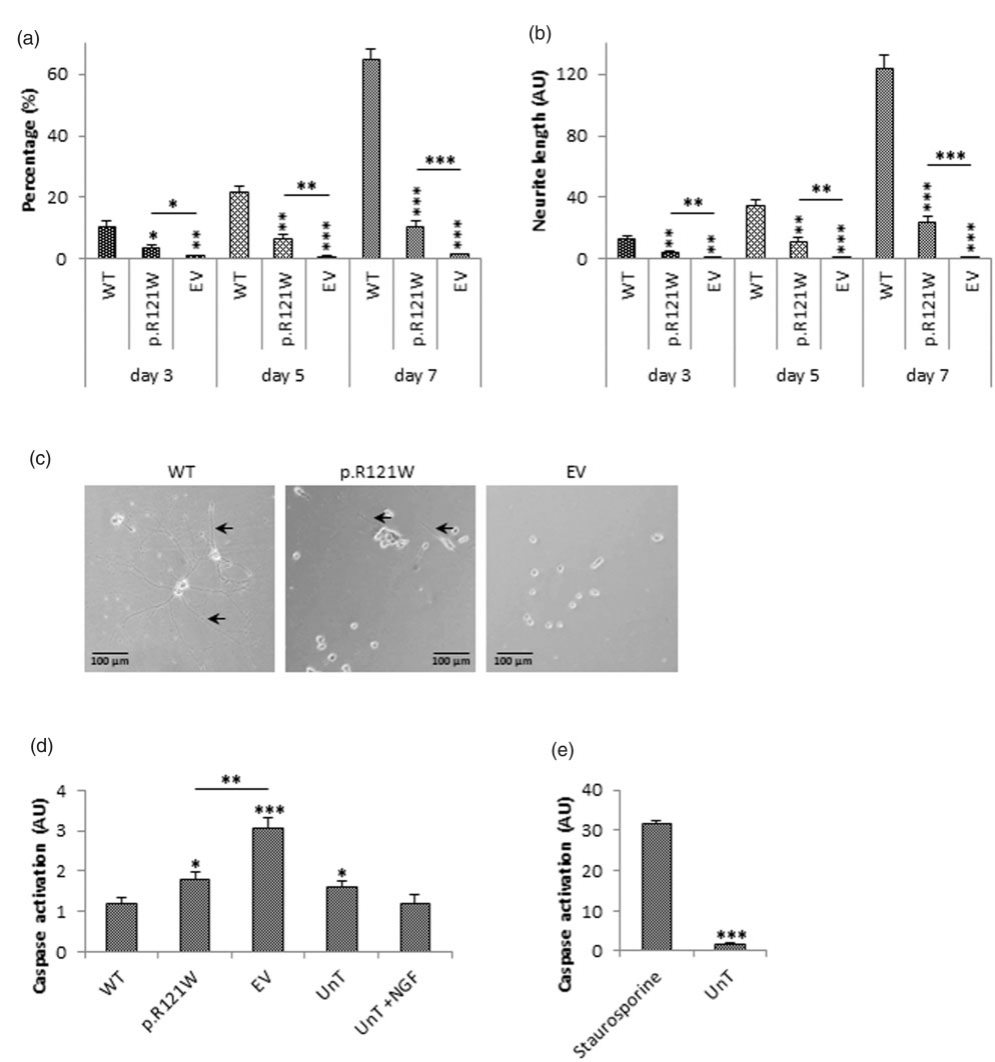
Differentiation and apoptotic activity of NGF. (a) PC12 cells were transfected, and the percentage of differentiated cells was quantified on days 3, 5 and 7. Sixty-five percent of wild-type transfected cells were differentiated by day 7, whereas only 10% of p.R121W NGF cells were differentiated. Cells transfected with empty vector did not show any differentiation. A neurite was identified as being more than twice the cell body length. (b) Average neurite length per cell was also quantified on days 3, 5 and 7. Average neurite length was 124.1 AU in wild-type transfected cells on day 7, whereas average length was only 23.7 AU in p.R121W cells. Empty vector transfected cells did not have any extensions. (c) Representative images from three independent experiments on day 7 are shown. Black arrows indicate neurites. Statistical differences between wild-type and mutant NGF on each day are indicated as **p<0.01 or ***p<0.001 (one-way ANOVA, followed by Tukey’s post hoc test). (d) To investigate if p.R121W had any apoptotic effect, caspase activity in PC12 cells transfected with the constructs or treated with 100 ng/ml NGF as the positive control was determined. Wild-type NGF transfected cells did not induce caspase activity and was similar to the positive control. Cells transfected with p.R121W had a slightly higher level of caspase activity. Cells transfected with empty vector had the highest level of caspase activity. As empty vector-transfected cells had the highest level of caspase activity, it suggests that transfection itself caused apoptosis, and neither wild-type nor p.R121W NGF induced apoptosis. The *x-*axis represents cells transfected with WT NGF, p.R121W and empty vector transfected cells or untransfected cells (UnT) or with untransfected cells treated with exogenous NGF (UnT+NGF). (*NTRK1, p75* and *sortilin* were equally expressed in non-transfected, wild-type NGF transfected and p.R121W mutant transfected PC12 cell; data not shown). Statistical differences between wild-type and the other treatment groups are indicated as *p<0.05, *p<0.01, ***p<0.001 (one-way ANOVA, followed by Tukey’s post hoc test). (e) Treatment of PC12 cells with staurosporine, an agent that is known to cause apoptosis, showed high levels of caspase activation. Statistical differences are indicated as ***p<0.001 (Student’s *t-*test). Error bars represent standard error of the mean, n = 3. WT: wild-type; EV: empty vector; UnT: untransfected.

Next, the pro-differentiation activities of tagged wild-type NGF and p.R121W NGF were determined. Transfected PC12 cells were cultured for seven days post-transfection, and percentage of differentiated cells and neurite outgrowth was quantified on days 3, 5 and 7 (Figure 4(a) to (c)).

On day 3, 10% of cells transfected with tagged wild-type NGF were differentiated, and average neurite length was 13.5 AU. 3.8% of cells transfected with p.R121W NGF were differentiated and average neurite length was 4.3 AU, which was significantly lower than the tagged wild-type effect (p < 0.05). Only about 1% of cells transfected with empty vector were classified as differentiated, and the average neurite length was 1 AU.

On day 5, the number of differentiated tagged wild-type NGF-transfected cells increased 2.5-fold to 25.6% and average neurite length increased to 34.6 AU. The percentage of differentiated p.R121W-transfected cells increased to 6.5% and average neurite length increased to 11.1 AU, which were significantly lower than the wild-type results (p < 0.001) but significantly higher compared to empty vector-transfected cells (p < 0.01).

On day 7, 64.5% of tagged wild-type NGF-transfected cells were classified as differentiated, and the average neurite length was 124.1 AU. The percentage of differentiated p.R121W-transfected cells increased to 10.5%, and average neurite length was 23.7 AU. This was lower than cells transfected with tagged wild-type NGF (p < 0.001) but was higher than empty vector-transfected cells (p < 0.001). These studies indicate that p.R121W NGF had residual, but much reduced, neurotrophic activity when compared to wild-type NGF.

### Apoptotic activity of proB form

As many studies in the literature have indicated that proNGF is an apoptotic molecule, we next assessed the levels of caspase activity in transfected cells. PC12 cells (known to express both p75 and sortillin^7^) transfected with tagged wild-type NGF did not induce apoptosis, and the level of caspase activity was 2.5-fold lower than in empty vector-transfected cells (p < 0.001) as shown in Figure 4(d). This suggests that transfection itself caused cell death but this effect could be rescued by the protective effects of wild-type NGF. Cells transfected with p.R121W NGF had a level of caspase activity that was significantly lower than empty vector-transfected cells (p < 0.01) but significantly higher than wild-type NGF-transfected cells (p < 0.05). This indicated that p.R121W also had a protective effect but one that was not as effective as wild-type NGF. We assessed our apoptosis and caspase activation system with a positive control apoptotic molecule staurosporine; all our results are very different from the large effects mediated by staurosporine (Figure 4(e)). Together these results show that p.R121W mutation did not significantly induce apoptosis in this model.

## Discussion

In this study, we report a novel missense mutation found in a patient with a HSAN5 phenotype. We have determined the effect of this mutation on NGF function and have shown the intermediate proNGF cleavage form proB is not apoptotic but rather has reduced neurotrophic activity compared to mature NGF and in contrast to the effect of full-length proNGF.

The HSAN5 patient p.R121W mutation did not affect the localisation nor the secretion of NGF. In our system, immunoblotting lysate and media showed that tagged wild-type NGF was present mainly as mature NGF and no intermediate cleavage product could be seen, indicating that cleavage of proNGF was very efficient. On the other hand, p.R121W was mainly present as the proA form and proB form in the lysate which most likely correspond to cleavage at positions -74, -73 and -43, -42, respectively. ProB was the only form secreted by p.R121W-transfected cells and interestingly no mature NGF could be observed indicating that the mutation completely abolished cleavage.

The ability of the proB form to induce downstream signalling was then investigated. This cleavage intermediate could not induce significant autophosphorylation of TRKA but could induce phosphorylation of MAPK. This may have been because the levels of NGF are already low in the conditioned media, and as PC12 cells have endogenous TrkA, the effect observed on wild-type TRKA-GFP may have been masked. Thus, the level of autophosphorylation may be beyond the sensitivity of the assay, but as signalling cascades are amplified, it is sufficient to cause detectable levels of downstream events such as MAPK phosphorylation. Furthermore, neurite outgrowth experiments showed that the proB form had neurotrophic activity, although it was five-fold lower than wild-type NGF. As proNGF in some studies has been demonstrated to have an apoptotic role, the apoptotic activity of the proB form was also investigated. However, the proB form did not induce apoptosis. These results indicate that p.R121W is pathogenic through its inability to induce differentiation, rather than its ability to induce apoptosis.

Previous studies have demonstrated that a two-fold reduction in TRKA activity caused by *NTRK1* mutations is sufficient to cause a reduction in neurite growth and HSAN4.^22^ This suggests that a five-fold reduction in differentiation activity is almost certainly insufficient to induce proper development of nociceptors. The patient with this NGF mutation did not present with a less, or more severe, HSAN5 phenotype. This suggests that partial NGF activity is inadequate to drive neuronal differentiation, providing further evidence that there is an optimal level of NGF-TRKA signalling required for neuronal development.

Despite the general consensus that proNGF is an apoptotic agent,^7,11^ the data presented here do not support an apoptotic role for the intermediate cleavage forms of proNGF, as the mutation p.R121W which prevented cleavage to mature NGF did not induce apoptosis. This result is consistent with a number of studies in the literature. For example, three studies have shown that cleavage-resistant p.R121G proNGF interacted with TRKA and caused downstream signalling and neurite outgrowth with a five-fold reduction in activity.^9,27,28^

The discrepancy between the apoptotic and neurotrophic effects of the different types of cleavage-resistant proNGF molecules may be due to the differences in the cleavage mutations. The neurotrophic cleavage-resistant form of proNGF used by the Fahnestock and Dawbarn groups used the mutation p.R121G which involves the same amino acid R121 as our p.R121W mutation.^9,27,28^ The p.R121G form also resulted in cleavage at the other sites and production of proB.^7^ The apoptotic proNGF used by the Hempstead and Neet groups contains mutations at all cleavage sites and thus only full-length proNGF is formed^7,11,28^ Consequently, compared to full-length proNGF, the shorter proB form may have a higher affinity for TRKA rather than p75NTR or sortilin, and thus is capable of inducing neurite outgrowth and not apoptosis. Further work is necessary to determine the binding affinities of the proB form of NGF with sortilin, p75NTR and TRKA. In our experiments, there was no evidence that proNGF or proA-NGF was secreted; however, proB-NGF was easily detected and secreted in biologically significant amounts; why proB-NGF is secretable is also worthy of future study. Together this investigation of a rare NGF missense mutation supports the hypothesis that the HSAN5 phenotype is caused by an insufficiency of mature NGF, rather than the effects of induced apoptosis on developing nociceptors.
